# A systematic review and standardized clinical validity assessment of genes involved in female reproductive failure

**DOI:** 10.1530/REP-21-0486

**Published:** 2022-03-29

**Authors:** Ludmila Volozonoka, Anna Miskova, Liene Kornejeva, Inga Kempa, Veronika Bargatina, Linda Gailite

**Affiliations:** 1 Scientific Laboratory of Molecular Genetics, Riga Stradins University, Riga, Latvia; 2 E. Gulbja Laboratory, Riga, LV-1006, Latvia; 3 Department of Obstetrics and Gynaecology, Riga Stradins University, Riga, Latvia; 4 Riga Maternity Hospital, Riga, LV-1013, Latvia

## Abstract

Genetic testing is becoming increasingly required at almost every stage of failed female reproduction/infertility. Nonetheless, clinical evidence for the majority of identified gene–disease relationships is ill-defined, thus leading to difficult gene variant interpretation and poor translation of existing knowledge into clinics. We aimed to identify the genes that have ever been implicated in monogenic female reproductive failure in humans and to classify the identified gene–disease relationship pairs using a standardized clinical validity assessment. A PubMed search following PRISMA guidelines was conducted on 20 September 2021 aiming to identify studies pertaining to genetic causes of phenotypes of female reproductive failure. The clinical validity of identified gene–disease pairs was assessed using standardized criteria, counting whether sufficient genetic and experimental evidence has been accumulated to consider a single gene ‘characterized’ for a single Mendelian disease. In total, 1256 articles were selected for the data extraction; 183 unique gene–disease pairs were classified spanning the following phenotypes: hypogonadotropic hypogonadism, ovarian dysgenesis, premature ovarian failure/insufficiency, ovarian hyperstimulation syndrome, empty follicle syndrome, oocyte maturation defect, fertilization failure, early embryonic arrest, recurrent hydatidiform mole, adrenal disfunction and Mullerian aplasia. Twenty-four gene–disease pairs showed definitive evidence, 36 – strong, 19 – moderate, 81 – limited and 23 – showed no evidence. Here, we provide comprehensive, systematic and timely information on the genetic causes of female infertility. Our classification of genetic causes of female reproductive failure will facilitate the composition of up-to-date guidelines on genetic testing in female reproduction, the development of diagnostic gene panels and the advancement of reproductive decision-making.

## Introduction

Female infertility is an ongoing global challenge which has significant medical, social and psychological implications. It is estimated to affect as much as 16.2% of women in certain countries (Singh 2004, Inhorn & Patrizio 2015, Maddirevula *et al.* 2020). Clinical recognition of the genetic causes of female reproductive failure using increasingly advanced genetic technologies is a major challenge for reproductive medicine in the 21st century.

Genetic disorders (chromosome changes and monogenic disorders) are responsible for around 5 –10% of female infertility or subfertility (Toth *et al.* 2019). However, it is difficult to estimate the genetic component of the wider umbrella term of female reproductive failure which spans impaired ability to conceive through carrying a term ‘pregnancy’. The identification of genetic causes of female infertility began in the late 1950s when the aetiology of Turner syndrome was discovered by means of karyotyping (Ford *et al.* 1959) and continues to this day aided by the development of novel molecular techniques and technological advancements. Female reproductive health is very much dependent on the correct functioning of the hypothalamic–pituitary–gonadal (HPG) axis (Guerri *et al.* 2019). The main actors of HPG axis include gonadotrophin-releasing hormone, gonadotropins–luteinizing hormone and follicle-stimulating hormone, as well as steroid hormones synthesized in the adrenal cortex and the gonads. Hormonal regulation of the HPG axis is essential for the development, maturation and release of an oocyte – functional unit of the ovary that is required for successful propagation of the species, as well as for female secondary sexual characteristics and for supporting pregnancy (Chengalvala *et al.* 2006, Maggi *et al.* 2016). Consequently, widely acknowledged monogenetic causes of female reproductive failure include genes functioning within the HPG axis, for example, pathogenic *KISS1R* variants causing hypogonadotropic hypogonadism, *FMR1* and *FSHR* variants leading to premature ovarian insufficiency and *CYP17A1*causing congenital adrenal hyperplasia (Kim *et al.* 2014, He *et al.* 2019, Moalla *et al.* 2019, Villa *et al.* 2021).

With the advent of next-generation sequencing (NGS) (including gene panels, whole-exome and whole-genome sequencing), it is now possible to understand the genetic underpinnings of female infertility at a higher resolution. Indeed, numerous genes have been discovered to be involved in the proper functioning of female reproduction, but challenges exist with proving their causality of certain phenotypes and the subsequent translation of this information into clinical practice (Mallepaly *et al.* 2017). It is not uncommon for review articles to list genes without a solid implication into human phenotype and without clinical validity assessment as causative ones. Unfortunately, any disease/condition-associated gene list without systematic analysis and clinical validity assessment is arbitrary. Acknowledging known genes, candidate genes and risk factors in the same context can result in overemphasis of particular genes in the scientific and medical literature and consequently, the malicious practice of incorporating such genes into testing panels in clinics. The diagnosis of a genetic disease can be a challenge and is contingent upon a robust understanding of the disease’s molecular aetiology (gene structure, function, previously identified variants, disease mechanism) in addition to a comprehensive clinical knowledge of the patient’s medical history (Ellard *et al.* 2020). A major challenge for diagnostic laboratories is interpreting the clinical validity of a gene–disease association, defined as ‘the determination that a particular disease is truly caused by variants in a particular gene and that the specific variant that has been detected is indeed pathogenic’, that is, whether a gene is ‘characterized’ (Biesecker & Green 2014, Smith *et al.* 2017). Ideally, only characterized genes should be included in diagnostic panels, as recommended by both the European Society of Human Genetics (Matthijs *et al.* 2016) and the American College of Medical Genetics and Genomics (Rehm 2013).

There is a growing importance of molecular testing in reproductive medicine (Cariati *et al.* 2019). In human reproduction, genetic tests should be carried out for various purposes: (a) to identify the cause of reproductive failure, (b) to identify genetic diseases transmissible to offspring, (c) to provide direction towards the most appropriate assisted reproductive technology (ART) and (d) to estimate the possible risk of infertility-associated co-morbidities.

Reproduction societies have guidelines for different female infertility phenotypes, with the majority of them acknowledging the genetic aetiology of a specific condition, for example, for POI, hypogonadotropic hypogonadism (Boehm *et al.* 2015, Webber *et al.* 2016). Unfortunately, genetic testing strategy, if advised, is often outdated, vague or disregarded unless there is convincing evidence suggesting a genetic pathology, that is presence of the syndromic form of the condition. There are currently no authority guidelines or committee opinions offering a clear integrative genetic testing scheme for female reproductive failure/infertility. Consequently, very few specific tests are routinely recommended to investigate the presence of chromosomal disorders and especially single-gene defects related to patients’ clinical phenotypes (Cariati *et al.* 2019). In 2002, Foresta and colleagues recommended karyotyping and molecular testing of *FMR1*, *ANOS1* and *CFTR* in their ‘Guidelines for the appropriate use of genetic tests in infertile couples’ (Foresta *et al.* 2002). Despite the passing of almost two decades since these guidelines were published, nothing much has changed. Specifically, in 2017, Stuppia and Gatta recommended karyotyping and only *FMR1* assessment (Stuppia & Gatta 2017), while in 2019, Toth and colleagues recommended karyotyping, *FMR1* and *CYP21A2* testing, as well as congenital hypogonadotropic hypogonadism gene panel testing (Toth *et al.* 2019). It is worth mentioning that *CFTR* testing is recommended to identify carrier patients with an increased risk of having children with cystic fibrosis, while *FMR1* premutation testing serves two purposes – to identify the cause of infertility and to indicate females with an increased risk of having progeny with fragile X syndrome (Foresta *et al.* 2002). Of note, there is currently no evidence of X-linked *ANOS1* variants causing hypogonadotropic hypogonadism in females, as in males, ANOS1 variants display a complete penetrance with severe expressivity of the condition (Dodé & Hardelin 2010, Neocleous *et al.* 2020).

Taking all the aforementioned information into consideration, we designed this study to achieve two goals. First, we conducted a systematic literature search in order to extensively identify genes related to reproductive failure/infertility in women. Secondly, using a standardized framework developed by Smith and colleagues (Smith *et al.* 2017), we evaluated the clinical validity of the identified gene–disease pairs. Our work resembles a similar effort successfully made for male infertility phenotypes (Oud *et al.* 2019, Houston *et al.* 2021).

## Materials and methods

### Systematic literature analysis: search strategy and study selection

To minimize bias in the data collection, the literature search was conducted according to the Preferred Reporting Items for Systematic Reviews and Meta-Analysis (PRISMA) guidelines (Moher *et al.* 2009). The screening strategy retrieved studies aimed at identifying a direct genetic cause for failed female reproduction, including pre-gonadal, gonadal, post-gonadal, as well as eugonadal phenotypes. The search was performed in PubMed on several occasions (the last search was performed on 20 September 2021) using various female reproductive failure and genetics-related keywords (please see full search strategy, inclusion and exclusion criteria in Supplementary File 1A, see section on supplementary materials given at the end of this article). Severe genetic conditions where infertility is a minor manifestation of the condition were excluded, leaving only those syndromic forms of reproductive failure where it is one of the major manifestations of the syndrome. Initially, titles and abstracts of the identified articles were pre-screened for relevance. Next, full-text articles were assessed and genes were extracted. Individual reasons for rejection were documented. Additionally, 45 review articles published from 2019 onwards were screened to identify potentially missed genes implicated in the question of study (study-level bias assessment). Ethical approval was not sought for the current systematic literature analysis and gene–disease clinical validity assessment as data is synthesized from previously published studies in which informed consents were obtained by primary investigators and no sensitive patient data are disclosed herein.

### Clinical validity assessment of gene–disease pairs

We exploited standardized criteria developed by Smith and colleagues to assess the clinical validity of identified gene–disease associations (Smith *et al.* 2017). This protocol was in turn based on the framework developed by the Clinical Genome Resource (Strande *et al.* 2017). Gene–disease pairs assessment utilizes a points system, counting whether sufficient genetic and experimental evidence has been accumulated to consider a single gene ‘characterized’ for a single Mendelian disease. Evidence collection accuracy is facilitated through systematization and comprehensive documentation. Genetic evidence considers the pathogenicity of variants, number of patients and number of replication studies. Pathogenicity of gene variants is evaluated using the guidelines of the American College of Medical Genetics and Genomics (ACMG) (Richards *et al.* 2015). Experimental evidence considers gene function/expression, disruption experiments and model organisms. The data for each gene record are primarily gathered from the particular article describing gene–disease pair, and missing information is searched upon genetic databases (e.g. OMIM, Protein Atlas and MGI) and/or additional publications. Based on the points collected, a characterized gene–disease pair obtains a certain validity strength: moderate, strong or definitive. When there are no enough data to support the gene–disease relationship, a mark of ‘limited evidence’ or ‘no evidence’ leaves the gene, a candidate for the particular phenotype. The curation protocol is detailed in Supplementary File 1B. We introduced only one modification to Smith* et al*.’s system – we marked the gene record from the certain publication as ‘unable to classify’ if the described variant was classified as (likely) benign according to the ACMG guidelines and did not process it any further.

During each gene’s classification process, an additional PubMed search was performed in order to identify all possible replication studies (marked as ‘found additionally’; Supplementary Table 2) (study-level bias assessment). We extracted all gene records using the latest Human Genome Organization nomenclature. We also extracted the genetic technology used to analyse the gene, reported and known (OMIM data) inheritance pattern of a disease and described disease cases (syndromic or isolated and sporadic or familial). A proportion of genes (*n* = 10) was classified by the two scientists; all inconsistencies were discussed until a consensus was reached (outcome-level bias assessment).

### Characterization of genes with a sufficient level of evidence

Gene–disease dyads classified as moderate, strong or definitive were grouped into disease classes drawn across the HPG axis. We also checked whether any of characterized genes have been approved as clinically valid for male infertility phenotypes by comparing our data with those of Oud* et al*. who utilized the same curation framework to identify genes confidently linked to human monogenic male infertility (Oud *et al.* 2019, Houston *et al.* 2021). The tissue expression pattern of genes was assessed using data from the mRNA Human Tissue Atlas (https://www.proteinatlas.org).

## Results and discussion

Our publication search, conducted according to the PRISMA guidelines, aimed to identify articles focusing on the genetic causes of female reproductive failure (Supplementary Tables 1 and 2). Furthermore, the identified gene–disease dyads were assessed for clinical validity based on Smith* et al*.’s framework (Smith *et al.* 2017).

### Publication and gene analysis

**Figure 1:**
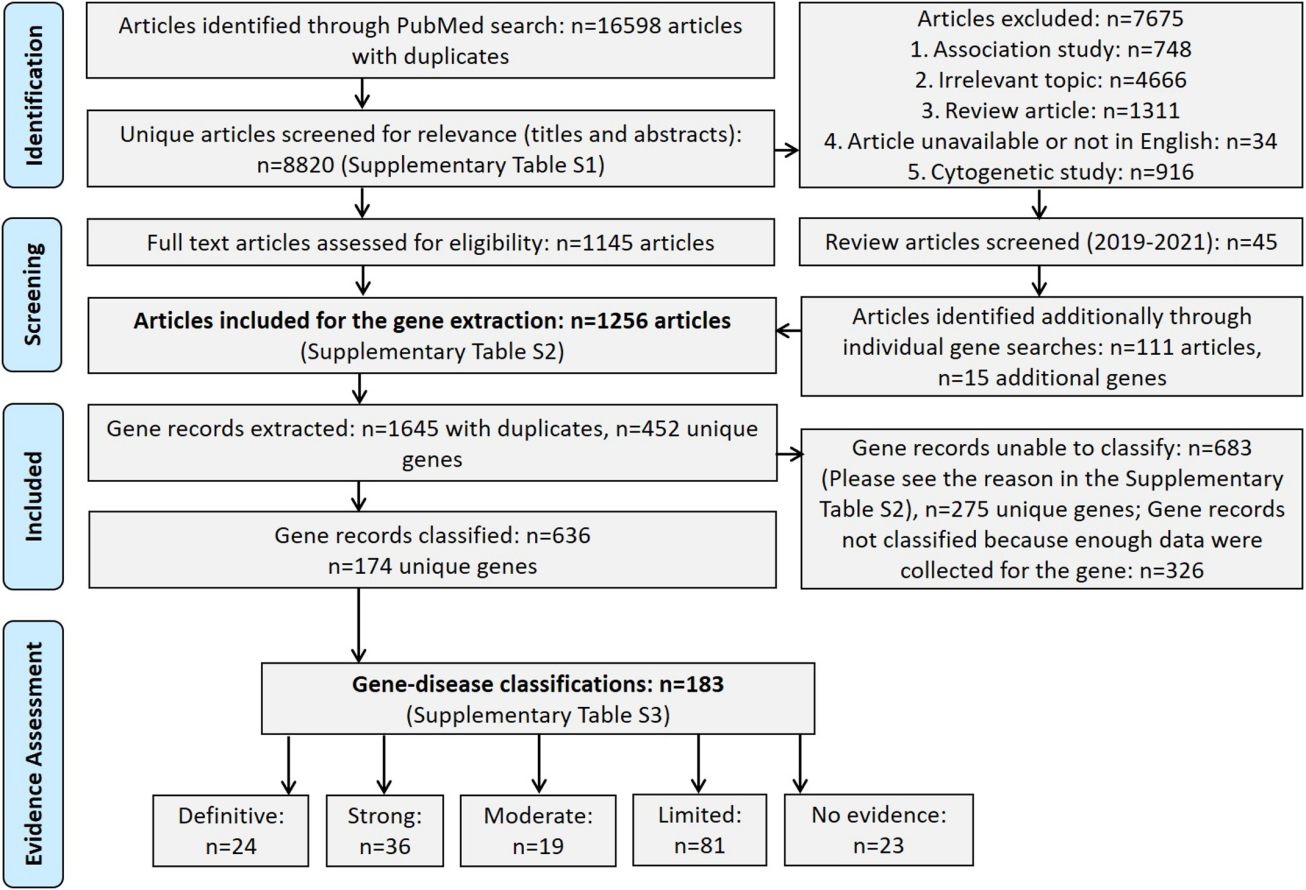
PRISMA flowchart detailing our publication identification strategy and gene classification outcomes for female reproductive failure.

**Figure 2:**
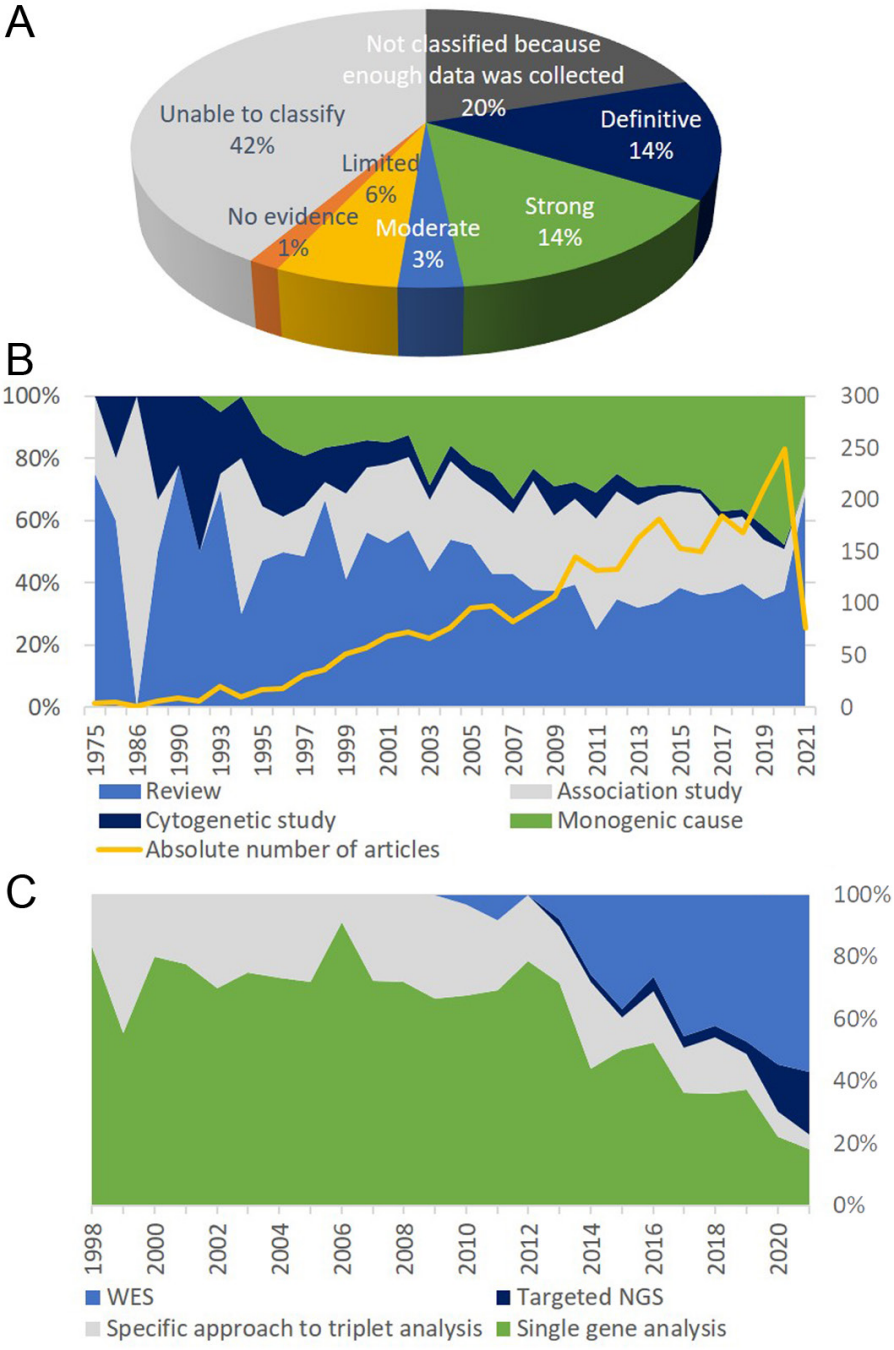
Article and gene analysis focusing on female reproductive failure derived from our PubMed search up until 20 September 2021 (for details, please refer to Supplementary Tables 1, 2 and 3). (A) Outcomes for the articles selected for the gene extraction (data from Supplementary Tables 2 and 3). (B) Types of genetic articles on female reproductive failure published over the years (data from Supplementary Tables 1 and 2). (C) Genomic technologies used to analyse genes of female reproductive failure; percentage indicates the fraction of articles identified in a particular year (data from Supplementary Table 2). WES, whole-exome sequencing, NGS, next-generation sequencing.

In total, 8820 articles were screened for relevance, and out of those, 7675 were excluded (please see rejection reasons in Supplementary Table 1); 1256 scientific articles were selected for the gene extraction, from which 1645 gene records with duplicates (here and further in the text particular gene assessed in certain study is referred as ‘gene record’) or 452 unique genes were identified (Fig. 1). Of those, 683 gene records or 42% (Fig. 2A) could not be classified due to unmet criteria to be classified or due to unsuitability of the classification system to classify certain relationship classes (e.g. association studies; please see individual rejection reasons in Supplementary Table 2); 275 unique genes could not be classified (please see Supplementary Table 4).

Overall, 636 gene records or 174 individual genes were classified, ultimately leading to 183 unique gene–disease classifications as variants in some genes are causing more than one condition. Twenty-four gene–disease pairs showed definitive evidence, 36 – strong, 19 – moderate, 81 – limited and 23 showed no evidence (please see Supplementary Table 2 for the detailed gene records analysis and Supplementary Table 3 for the complete gene curation process). Genes having at least moderate level of evidence are considered classified and with confidence can be used in clinics (Table 1). The genes most studied in association with female reproductive phenotypes were *FMR1* (145 records), *FOXL2* (59 records), *CYP17A1* (52 records), *FSHR* (48 records), *FGFR1* (42 records), *GNRHR* (39 records), *NLRP7* (38 records), *NR5A1* (27 records), *KISS1R* (26 records), *BMP15* (23 records) and *GDF9* (21 records) (Supplementary Table 3).

**Table 1:** Characterized female reproductive failure gene–disease pairs with at least moderate level of evidence.

HGNC gene name	OMIM gene phenotype identifiler	Reported inheritance pattern	Condition	Evidence level
*KISS1R*	614837	AR	Hypogonadotropic hypogonadism^*^	D
*FGFR1*	147950	AD	Hypogonadotropic hypogonadism^*^	D
*FGF8*	612702	AD	Hypogonadotropic hypogonadism^*^	M
*HS6ST1*	614880	AD,AR	Hypogonadotropic hypogonadism^*^	S
*TACR3*	614840	AD,AR	Hypogonadotropic hypogonadism^*^	D
*TAC3*	614839	AR	Hypogonadotropic hypogonadism^*^	S
*PROKR2*	244200	AD,AR	Hypogonadotropic hypogonadism^*^	D
*PROK2*	610628	AD,AR	Hypogonadotropic hypogonadism^*^	S
*GNRHR*	146110	AD	Hypogonadotropic hypogonadism^*^	D
*GNRH1*	614841	AD,AR	Hypogonadotropic hypogonadism^*^	S
*CHD7*	612370	AD	Hypogonadotropic hypogonadism^*^	D
*FSHB*	229070	AR	Hypogonadotropic hypogonadism	S
*POLR3B*	614381	AR	4H leukodystrophy associated with hypogonadotropic hypogonadism^*^	D
*POLR3A*	607694	AR	4H leukodystrophy associated with hypogonadotropic hypogonadism^*^	D
*POLR1C*	616494	AR	4H leukodystrophy associated with hypogonadotropic hypogonadism^*^	S
*PNPLA6*	215470	AR	BoucherNeuhäuser syndrome associated with hypogonadotropic hypogonadism^*^	S
*SOX10*	611584; 613266	AD	Waardenburg syndrome associated with hypogonadotropic hypogonadism^*^	S
*DCAF17*	241080	AR	Woodhouse–Sakati syndrome associated with hypogonadotropic hypogonadism^*^	S
*LEPR*	614963	AR	Obesity associated with hypogonadotropic hypogonadism^*^	S
*PROP1*	262600	AR	Combined pituitary hormone deficiency (CPHD) associated with hypogonadotropic hypogonadism^*^	D
*SEMA3A*	614897	AD,AR	Skeletal dysplasia associated with anosmia and hypogonadotropic hypogonadism^*^	M
*PSMC3IP*	614324	AR	POI	S
*NR5A1*	612964	AD	POI	D
*MCM8*	612885	AR	POI	S
*MCM9*	616185	AR	POI	S
*STAG3*	615723	AR	POI	S
*HFM1*	615724	AR	POI	M
*FSHR*	233300	AR	POI	D
*FOXL2*	110100	AD,AR	POI	S
*BMP15*	300510	XLD,XLR	POI	S
*GDF9*	618014	AR	POI	S
*FANCM*	618096	AR	POI	M
*NOBOX*	611548	AD,AR	POI	S
*SOHLH1*	617690	AR	POI	M
*MRPS22*	618117	AR	POI	M
*FMR1*	311360	XLD	POI	D
*NHEJ1*	NA	AD	POI	M
*AARS2*	615889	AR	Progressive leukoencephalopathy associated with POI^*^	S
*TWNK*	616138	AR	Perrault syndrome associated with POI^*^	M
*EIF2B2*	603896	AR	Ovarioleukodystrophy associated with POI^*^	M
*EIF2B5*	603896	AR	Ovarioleukodystrophy associated with POI^*^	D
*CLPP*	614129	AR	Perrault syndrome associated with POI^*^	M
*AIRE*	240300	AD,AR	Autoimmune polyendocrinopathy syndrome with POI^*^	S
*LARS2*	615300	AR	Perrault syndrome associated with POI^*^	S
*HARS2*	614926	AR	Perrault syndrome associated with POI^*^	S
*HSD17B4*	233400	AR	Perrault syndrome associated with POI^*^	M
*FOXL2*	608996	AD	Bplepharophimosis, epicanthus inversus and ptosis syndrome associated with POI^*^	D
*FH*	150800	AD	Hereditary leiomyomatosis and renal cell carcinoma associated with POI^*^	D
*POLG*	157640; 258450	AD,AR	Progressive external ophthalmoplegia associated with POI^*^	S
*SETX*	606002	AR	Oculomotor apraxia type 2 associated with POI^*^	M
*GGPS1*	619518	AR	Muscular dystrophy with hearing loss and POI (Perrault syndrome)^*^	M
*NUP107*	618078	AR	Ovarian dysgenesis	M
*FSHR*	608115	AD	Spontaneous ovarian hyperstimulation syndrome	S
*LHCGR*	238320	AR	Empty follice syndrome/luteinizing hormone resistance	S
*ZP1*	615774	AR	Empty follicle syndrome	D
*TBPL2*	NA	AR	Oocyte maturation defect	M
*TRIP13*	619011	AR	Oocyte maturation defect	M
*TUBB8*	616780	AD,AR	Oocyte maturation defect	D
*ZP3*	617712	AD,AR	Oocyte maturation defect	S
*ZP2*	618353	AD,AR	Oocyte maturation defect	S
*PATL2*	617743	AR	Oocyte maturation defect	D
*WEE2*	617996	AR	Oocyte fertilization failure	D
*TLE6*	616814	AR	Early embryonic arrest	S
*NLRP5*	NA	AR	Early embryonic arrest	S
*NLRP2*	NA	AR	Early embryonic arrest	S
*PADI6*	617234	AR	Early embryonic arrest	S
*CDC20*	NA	AR	Early embryonic arrest	S
*FBXO43*	NA	AR	Early embryonic arrest	M
*MEI1*	618431	AR	Hydatidiform mole	M
*KHDC3L*	614293	AR	Hydatidiform mole	D
*NLRP7*	231090	AD,AR	Hydatidiform mole	D
*CYP17A1*	202110	AR	Congenital adrenal hyperplasia 46,XX^*^	D
*POR*	613571	AR	Cytochrome P450 oxidoreductase deficiency^*^	S
*NR3C1*	615962	AD	Glucocorticoid resistance^*^	M
*CYP21A2*	201910	AR	Hyperandrogenism, non-classic type, due to 21-hydroxylase deficiency^*^	D
*HSD3B2*	201810	AR	Non-classical 3 beta-hydroxysteroid dehydrogenase deficiency^*^	S
*CYP11B1*	202010	AR	Non-classical congenital adrenal hyperplasia^*^	D
*WNT4*	158330	AD	Mullerian aplasia and hyperandrogenism (Mayer Rokitansky Kuster Hauser syndrome)^*^	S
*DICER1*	138800	AD	Multinodular goiter with or without Sertoli–Leydig cell ovarian tumors associated with virilization and amenorrhea^*^	S

^*^Syndromic form of the condition.

AD, autosomal dominant; AR, autosomal recessive; D, definitive; M, moderate; NA, not available;POI, premature ovarian insufficiency; S, strong; XLD, X-linked dominant; XLR, X-linked recessive.

Analysis of the publications focusing on female reproductive failure genetics over the years demonstrated that this area of research gained scientific interest in the late 1990s, coinciding with the development and wide adaptation of molecular techniques. The number of studies focusing on monogenic causes of impaired female reproduction has gradually increased, on the contrary, the relative number of cytogenetic studies has dropped down. A sharp increase in the proportion of review articles during the years 2020–2021 can be explained with the SARSCoV2 pandemic (Fig. 2B). Single-gene analysis (i.e. Sanger sequencing) was the main technique used to investigate the genetic causes of failed female reproduction, while NGS marked the dominating era of genomics around the years 2014 and 2015 (Fig. 2C), with whole-exome sequencing being the method of choice.

### Isolated vs syndromic forms of female reproductive failure

**Figure 3:**
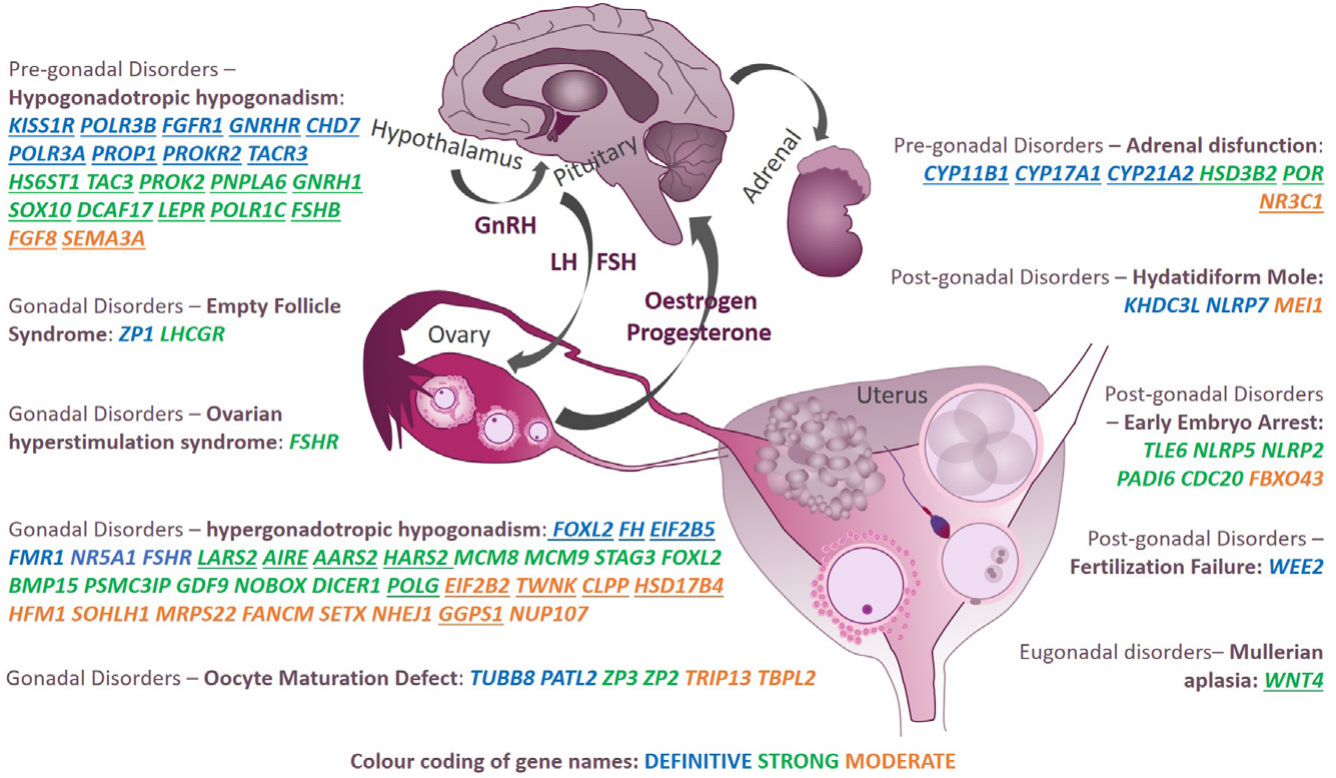
Genes at least moderately linked to a certain reproductive phenotype in females across different disease classes (pre-gonadal, gonadal, post-gonadal) reported in PubMed up until 20 September 2021. Underlined genes indicate syndromic forms of the reproductive phenotype. Genes in blue, green and orange indicate definitive, strong and moderate clinical validity classes, respectively. GnRH, gonadotropin-releasing hormone; LH, luteinizing hormone; FSH, follicle-stimulating hormone.

We depicted female reproduction-related gene–phenotype pairs having at least moderate clinical validity class based on their role within the HPG axis (Fig. 3) and recorded whether a particular gene causing reproductive phenotype was characterized as an isolated or syndromic form (avoiding severe syndromes, as described in Materials and methods) (Table 1). The phenotype having the most clinically valid gene associations was one of the most common infertility phenotypes – hypergonadotropic hypogonadism, which also includes POF/POI, occurring in ~1% of all females (Webber *et al.* 2016). Despite only 1% of POF cases being clinically syndromic forms (Qin *et al.* 2015), 14 out of 30 identified genes (46.6%) at least moderately linked to POF/POI were discovered in syndromic forms of the condition. Specifically, six genes cause Perrault syndrome characterized by POI and hearing loss (*TWNK*, *CLPP*, *LARS2*, *HARS2*, *HSD17B4*,* GGPS1*); three cause ovarioleukodystrophy or leukoencephalopathy with POI (*EIF2B2*, *EIF2B5*, *AARS2*); *FOXL2* causes blepharophimosis–ptosis–epicanthus inversus syndrome associated with POI; *FH* causes hereditary leiomyomatosis and renal cell carcinoma associated with POI; *AIRE* causes autoimmune polyendocrinopathy syndrome associated with POI; *POLG* causes progressive external ophthalmoplegia associated with POI and lastly, *SETX* causes oculomotor apraxia associated with POI (please see individual references in Supplementary Table 3).

The phenotype with the second highest number of characterized gene associations was hypogonadotropic hypogonadism being considered a pre-gonadal disorder. Twenty-one genes were at least moderately linked to specific syndromes associated with hypogonadotropic hypogonadism, Kallmann syndrome or a few isolated phenotype forms. While Kallmann syndrome is formally differentiated from normosmic hypogonadotropic hypogonadism, in practice, the transition is fluid (Boehm *et al.* 2015). The incidence of congenital hypogonadotropic hypogonadism in females is itself very rare (~1:40,000–125,000) (Meczekalski *et al.* 2013, Stamou & Georgopoulos 2018), making the identified relationships extremely rare entities.

Of particular interest was the specific phenotypes accessible only through the application of certain ART – without ART observable as idiopathic infertility – for example, empty follicle syndrome, oocyte maturation defect, fertilization failure, early embryonic arrest. Although these phenotypes had quite a limited number of characterized genes, the majority achieved either a strong or definitive level of clinical validity and were all found in non-syndromic forms.

The next group of disorders is associated with genes involved in steroid metabolism, leading to a syndromic form of infertility. While some of these genes primarily cause severe forms of disorders of sexual development/sex reversal, for example, congenital adrenal hyperplasia due to 21-hydroxylase deficiency, we only included phenotypes where infertility is a major expression of the disease. Thus, *CYP21A2*, *HSD3B2*, *CYP11B1*, *CYP17A1* and *POR* received definitive or strong levels of clinical validity and *NR3C1* received a moderate validity class.

A specific cause of failed pregnancy due to an abnormal growth of trophoblast – hydatidiform mole occurring in 1:600–1000 pregnancies in Western countries was at least moderately associated with three genes – was due to genes *KHDC3L* and *NLRP7*, both demonstrating a definitive validity class and *MEI1* receiving a moderate clinical validity class. Next syndrome occurring only in association with pregnancy – spontaneous ovarian hyperstimulation syndrome (sOHSS) – was shown to be caused by variants in *FSHR*. While *FSHR* is well known to cause non-syndromic POI, to date, very few cases of sOHSS have been associated with pathogenic variants in the rhodopsin-like serpentine domain of the gene, implicated in transduction of the activation signal to the interior of the cell (De Leener *et al.* 2006).

Lastly, severe form of reproductive system disorder, Mullerian aplasia also known as Mayer Rokitansky Kuster Hauser syndrome occurring in ~1:5000 women – characterized by the underdeveloped or absent vagina and uterus – was strongly associated with one gene *WNT4*(Petrozza *et al.* 1997).

Overall, out of 79 at least moderate associations, 37 gene–disease pairs (46.8%) were found in isolated phenotype forms, 33 (41.8%) were found in syndromic phenotype forms and 9 (11.4%) were found in both forms. As the majority of identified syndromic infertility forms are progressive, timely recognition of certain symptomatic manifestations gives an opportunity to undertake measures to manage patients’ reproductive potential.

### The question of inheritance pattern in female reproductive failure

At least 83 gene–disease associations out of 183 were found to be caused by single-allele disruptions of genes (i.e. dominant inheritance). Several are well known to develop in a dominant manner, for example, normosmic hypogonadotropic hypogonadism and Kallmann syndrome due to pathogenic/causative variants (mutations) in *CHD7* considered a mild allelic variant of CHARGE syndrome (Kim *et al.* 2008), *NR5A1* causing isolated POI and *FH* causing hereditary leiomyomatosis and renal cell carcinoma associated with POI. For blepharophimosis–ptosis–epicanthus inversus syndrome associated with POI, heterozygous *FOXL2* variants are known to be inherited from healthy fathers or as *de novo* events (Corrêa *et al.* 2010, Grzechocińska *et al.* 2019). Similarly, *TUBB8* is one of the rare genes with a proposed autosomal dominant inheritance pattern causing oocyte maturation defect as an isolated form of female infertility. In affected females, heterozygous *TUBB8* variants are inherited from unaffected fathers and exhibit a dominant-negative effect, indicating that *TUBB8* has a specific pathophysiological role in oogenesis but not in spermatogenesis. However, homozygous individuals have also been reported (Feng *et al.* 2016, Huang *et al.* 2017, Xing *et al.* 2020).

At the same time, there were a number of associations reported without a clearly established inheritance pattern. For example, it has long been considered that *BMP15* causes POI in an X-linked dominant manner with numerous articles reporting heterozygous variants as causative (Laissue *et al.* 2006, Kumar *et al.* 2017). However, it has recently been demonstrated that biallelic null mutations lead to the pathology and do not cause the phenotype in heterozygous mother carriers (Mayer *et al.* 2017, Rossetti *et al.* 2020). Similarly, heterozygous *NOBOX* variants exerting their effects through haploinsufficiency have been thought to lead to POI, but studies showing no difference in folliculogenesis between heterozygous *Nobox* mutant mice and WT mice do not support this (Lechowska *et al.* 2011, Li *et al.* 2017).

All such inconsistencies in the inheritance pattern are important to acknowledge, especially for phenotypes associated with reproduction, as it raises the question of how alleles causing infertility are inherited. These inconsistencies remind us that gaps currently exist in our understanding of the molecular and genetic pathophysiology of female reproductive failure and indicate the need for purposeful functional studies and perhaps testing and interpretation guidelines for particular genes. However, while we await these experimental data, the usage of existing *in silico* metrics for gene–disease mechanism interpretation can be helpful, for example, gene intolerance to haploinsufficiency or missense variation – pLI and Z-scores developed by the gnomAD consortium (Lek *et al.* 2016, Samocha *et al.* 2017, Betancur & Buxbaum 2020). Thus, for example, *NR5A1* and *FOXL2* obviously causing the phenotype in AD form have pLI scores of 0.99 and 0.88, respectively, demonstrating their intolerance to haploinsufficiency. In turn, with the dominant genes *TUBB8* and *FH* having low pLI and Z scores (*TUBB8* pLI = 0.02, Z = 1.86; *FH* pLI = 0.09, Z = 1.27), one might doubt their involvement in the disease; however, in this scenario, low scores can be explained by the fact that individuals with pathogenic *TUBB8* and *FH*variants are found in the apparently healthy general population as the associated phenotypes show themselves only in adulthood. Additionally, *NOBOX* and *BMP15* (X-linked gene) have low scores (*NOBOX* pLI = 0, Z = 0.48; *BMP15* pLI = 0, Z = 0.07), as in the general population, there are carriers of the gene variants since both genes cause the condition if disrupted on both alleles.

### Expression patterns of genes causing female reproductive failure and association with male infertility

Males and females have identical genetic information across most of their genomes but harbour many distinct sex-specific characteristics. Therefore, it is thought that the majority of sexually dimorphic traits are due to differential expression of genes that are present in both sexes. It has also been suggested that sex-specific expression genes are major contributors to the high incidence of infertility in men and women (Gershoni & Pietrokovski 2014, 2017). Hence, we were interested in comparing the identified set of characterized genes linked to female reproductive failure with the genes reported to cause reproductive failure in men. We looked for the data exploiting exactly the same gene–disease validity assessment system as we did (Oud *et al.* 2019, Houston *et al.* 2021).

Thus, 25 genes at least moderately linked to female reproduction phenotypes were also confidently linked to male infertility (Supplementary Table 3, column ‘Implication in male infertility’). While the majority of these genes cause Kallmann syndrome or isolated hypogonadotropic hypogonadism, there were a few interesting cases linking the overlapping biology of gametogenesis in both genders. For instance, *LHCGR* causing isolated empty follicle syndrome/luteinizing hormone resistance in females (validity class: strong) also causes isolated Leydig cell dysfunction with hypogonadism or precocious puberty in males (validity class: definitive). Further, *NR5A1* is known to cause 46,XX and 46,XY sex reversal also causes isolated POI in females (validity class: strong) and isolated spermatogenic failure in males (validity class: strong). Additionally, *FSHR* is responsible for POI or ovarian hyperstimulation syndrome in females (validity class: strong) and also causes hypergonadotropic hypogonadism in males (validity class: moderate). Of note, the expression of *LHCGR* and *FSHR* is primarily enriched in testis, as shown by the mRNA Human Tissue Atlas data (www.proteinatlas.org).

While gene expression databases have a huge impact on gene analyses and the understanding of diseases, they can lack expression information in specific tissues (e.g. oocytes, embryos) due to difficulties in acquiring these tissues. Attempts have been made to elucidate the transcriptome of human oocytes and blastocysts, with 1909 and 3122 genes being reported to be uniquely expressed in human MII oocytes and human blastocysts, respectively (Kakourou *et al.* 2013). Oocyte-specific genes included the zona pellucida glycoproteins – *ZP1*, *ZP2*, *ZP3* – causing non-syndromic oocyte maturation defect, and members of the transforming growth factor-beta superfamily, for example, *GDF9* and *BMP15*, causing non-syndromic premature ovarian failure, as shown through our data curation. Having human oocyte/embryo gene expression profiles in common gene expression databases may be of use for identifying dysregulation leading to infertility and aiming gene–disease curation process. However, at the current time, these shortcomings should be acknowledged and taken into consideration during analyses of phenotypes associated with reproduction.

### Limitations of the study

One of the limitations of this study is that only monogenic causes of failed female reproduction were assessed. Gene disruptions caused by chromosomal rearrangements (explaining approximately 2% of female reproductive failure cases (Schreurs *et al.* 2000) and at least 10% of all POF cases (Lakhal *et al.* 2010, Jiao *et al.* 2012)), gene copy number alterations and digenic inheritance were excluded. Another possible limitation of our gene extraction process is the existence of multiple phenotypes associated with failed female reproduction and their various denominations. We tried to include all known phenotypes and their associated genes by screening review articles; however, specific forms may have been omitted. Additionally, as substantial number of syndromes also exhibit fertility issues as a part of the phenotype, some rare syndromes may have been omitted. We also encountered a few articles on hypogonadotropic hypogonadism that did not report the gender of the studied patients, thus making it impossible to analyse the data.

Furthermore, we would like to highlight the difficulties experienced assessing *FMR1* premutation allele involvement in the development of premature ovarian failure/insufficiency (POF/POI), which is by far the most well-known genetic association with female subfertility. While the gene achieved the ‘definitive’ clinical validity class due to more than 24 patients being reported in the scientific literature, problems arose concerning the specificity of the pathogenic allele (i.e. triplet expansion) acting as a risk factor and its incomplete penetrance. In fact, only 15–25% of women with the *FMR1* premutation were found to be affected by POF and just 6.5% of women with POF carried the *FMR1* premutation (Sherman 2000). Moreover, experimental evidence on how the *FMR1* premutation allele leads to the phenotype is currently not completely clear (Friedman-Gohas *et al.* 2020, Rose & Brown 2020). Lastly, the researchers responsible for developing the framework have acknowledged difficulties in assessing alleles with incomplete penetrance (Smith *et al.* 2017).

### Importance of gene–disease clinical validity assessment

A major advantage of using Smith* et al*.’s gene–disease evaluation system (Smith *et al.* 2017) is the ability to continuously evaluate and expand the formed gene set whenever new clinical and scientific data are published. Thus, genes currently regarded as candidates (marked as ‘limited’ or ‘no evidence’) can go on to achieve characterized gene status or, conversely, be discounted from involvement in a particular phenotype’s development. Importantly, standardized clinical validity criteria are also useful for diagnostic exome interpretation, thus maximizing analytic sensitivity and specificity, reducing the number of variants of unknown significance and ensuring that patient results are interpretable (Smith *et al.* 2017).

Unfortunately, the practice of incorporating uncharacterized genes (including candidate genes and risk factors) into diagnostic panels or genetic testing of multifactorial phenotypes without known monogenic causes (e.g. polycystic ovarian syndrome, miscarriage) is not uncommon. In such cases, sequence data interpretation can be quite troublesome due to very little being known about the involvement of these uncharacterized genes in human pathology. This, in turn, might lead to missed true causative allele(s), futile expectations of patients and, in the worst scenario, adverse reproductive outcomes. For example, Eskenazi and colleagues analysed a number of genes in POI patients using NGS (Eskenazi *et al.* 2021). While the authors claimed that the genes were chosen on the grounds of being the ones most commonly identified in women with POI in previous studies, we were unable to uncover evidence for the association of *GPR3*, *EIF2S2*and* BHLHB9*with any reproductive phenotype. Indeed, the lack of an association between *GPR3* and POI has been reported previously (Kovanci *et al.* 2008, Zhou *et al.* 2010).

Often genes from pathways playing role in female reproduction, for example DNA damage response (Carroll & Marangos 2013, Martin *et al.* 2019), are considered popular candidates responsible for female reproductive failure. While these genes constitute the basis employed by the female germline to mitigate the impact of DNA damage during development and are important determinants of female fertility, the number of DNA damage response genes characterized for the phenotypes of female reproductive failure as clinically relevant currently is limited. We have identified eight DNA damage response genes that met the criteria to be classified. *WNT4* received a strong, *FANCM* – a moderate clinical validity class, whereas *NBN*, *MRE11*, *FOXO3*, *INSR*, *FANCL* and *BRCA2* had limited or no evidence to be characterized.

Thus, we want to emphasize again that gene selection process not based on a proper clinical validity assessment of the genes is arbitrary and should be disregarded for clinical purposes. Increasing the collaboration between clinicians and biomedical scientists involved in diagnostics and basic research should give rise to an easily accessible and up-to-date clinical data resource for female reproduction phenotypes and genotypes. In the absence of collaboration, researchers should submit their results to existing public genomic databases, for example, Varsome (https://www.varsome.com/) (Kopanos *et al.* 2019).

### Clinical genetic testing in failed female reproduction

As demonstrated by our systematic analysis and clinical validity evaluation of the genes involved in the development of female reproductive failure, significant success has been achieved during the last few decades in deciphering the molecular and genetic basis of this subject matter. These data provide extensive, systematic and timely information on the monogenic causes of impaired female reproduction. Genes mentioned here (Table 1) can be with confidence incorporated into clinical testing in females with certain phenotypes of failed reproduction, as anticipated improving the diagnostic yield for underlying genetic causes of infertility, which have been defined as unexplained until now (Capalbo *et al.* 2021).

Maddirevula with colleagues attempted to analyse exomes of primary infertility and recurrent pregnancy loss patients (*n* = 75) (Maddirevula *et al.* 2020). NGS revealed pathogenic variants in five known genes – *NLRP5*, *TLE6*, *NLRP7*, *ZP1* and *FSHR*, resulting in a diagnostic yield of 6.6%, which is quite high considering that there are no monogenic associations for recurrent pregnancy loss. It should be expected that for well-phenotyped patient cohorts, diagnostic yield may be higher. While it is too early to apply exome analysis for a female reproductive failure in a diagnostic setting, targeted assays including characterized genes could be reliably implemented into reproductive clinics’ workup. However, the advantage of sequencing exomes in the era of genomics when new disease genes are discovered regularly at an increasing pace is inarguable. In such scenario, targeting can be made *in silico* from the exome data allowing for a more versatile gene list adaptation, simultaneous testing of the patients spanning different female reproductive failure phenotypes and importantly reanalysis of the existing data. Apart from shorter turnaround time single NGS test saves financial resources, cost-effectiveness of >550% has been demonstrated when using NGS for female and male infertility evaluation compared to multiple genetic tests (Patel *et al.* 2018).

Furthermore, the clinical utility of such testing lies in the opportunity to opt for patient-tailored ARTs. Patients deserve realistic expectations of their reproductive status. For example, patients with *WEE2*variants causing oocyte fertilization failure were subjected to a decade of unsuccessful fertility treatments (Biswas *et al.* 2021). As demonstrated by our study, the proportion of genes involved in female reproductive failure also causes male infertility, this has important implications for expanded family screening and counselling. Additionally, knowledge of the genetic causes of infertility in parents will provide information on the risk of transmitting the same reproductive phenotype to the next generation, as well as offspring’s risk of developing infertility later in life (Capalbo *et al.* 2021).

We believe that in the near future, the data of this study together with updated best practice guidelines and proper genetic counselling will provide an unprecedented opportunity to increase the number of positive diagnoses and patient-tailored ARTs usage, thus taking the overall performance of reproductive medicine to a whole new level.

**Statements on genetic testing in failed female reproduction**

- The composition of genetic testing panels requires a clear distinction of the differences between causative genes, risk factors and genes only showing a positive association with certain reproductive failure phenotypes.
- The interpretation of sequence variants should follow existing guidelines (e.g. those of Richards *et al.* (2015)), taking into account disease inheritance patterns and mutational mechanisms.
- A patient’s clinical genetic diagnosis can be made solely from characterized genes with an established gene–disease clinical validity association; variants in genes with limited or no evidence should be interpreted with great caution.
- Effort should be made in research and clinics to elucidate the inheritance patterns of putative causative alleles in female reproductive failure, at least through analyses of parental loci.

## Conclusions

Out of 1645 gene records studied in relation to failed female reproduction/infertility, we identified 79 gene–disease pairs at least moderately linked to certain isolated or syndromic phenotypes: hypogonadotropic hypogonadism, ovarian dysgenesis, premature ovarian failure/insufficiency, ovarian hyperstimulation syndrome, empty follicle syndrome, oocyte maturation defect, fertilization failure, early embryonic arrest, recurrent hydatidiform mole, adrenal disfunction and Mullerian aplasia. Thus, 77 unique genes out of 452 (17%) implicated in female reproductive failure in literature can be assigned evidence strong enough to be used as diagnostic markers in clinics. Additionally, we identified 101 genes as candidates with only limited or currently no evidence for involvement in failed female reproduction. The data provided here provide a basis for continual gene curation in female reproductive failure over the years to come.

## Supplementary Material

Supplementary Material

Supplementary Table S1. Unique articles screened for relevance (titles and abstracts).

Supplementary Table S2. Gene extraction.

Supplementary Table S3. Gene curation.

Supplementary Table S4

## Declaration of interest

The authors declare that there is no conflict of interest that could be perceived as prejudicing the impartiality of the research reported.

## Funding

This work did not receive any specific grant from any funding agency in the public, commercial, or not-for-profit sector.

## Data availability statement

All data are incorporated into the article and its online Supplementary material.

## Author contribution statement

Conceptualization: L V, A M, L K, L G; Data curation: L V, L K, V B; Visualization: L V; Supervision: A M, I K, L G. All authors made substantial contributions to the editing of the manuscript.
