# Supplementary material for: A systematic review and standardized clinical validity assessment of genes involved in female reproductive failure
Source: Reproduction. 2022 Mar 29;163(6):351–63. doi: 10.1530/REP-21-0486 (PMC9066658; doi:10.1530/REP-21-0486)
Supplement: Supplementary Material [file supplementary_material.pdf]

Supplementary File 1.

A) PubMed search, study inclusion/exclusion criteria

The search was performed in PubMed on several occasions (the last search was performed on 20 September 2021) using the following strategy and keywords: ((female infertility OR female reproduction OR pregnancy loss OR miscarriage OR reproductive loss OR empty follicle syndrome OR meiotic arrest OR premature ovarian failure OR premature ovarian insufficiency OR hypogonadotropic hypogonadism OR hypergonadotropic hypogonadism OR hydatidiform mole OR oocyte maturation defect OR early embryo arrest OR fertilization failure [Title/Abstract]) AND (gene OR genetics OR mutation OR variant [Title/Abstract])) AND “human”.

The inclusion criteria were as follows: study published in a peer-reviewed journal; study presenting original data; study focusing on identifying an underlying genetic cause for female reproductive failure. Only human studies were included.

The exclusion criteria were as follows: study investigating non-coding DNA, mitochondrial DNA, HLA region, ribosomal DNA, microbiome, epigenetics; genetic study not aimed at identifying a direct genetic cause of the condition (genetic association, genetic risk, expression study); study analysing chromosomal aneuploidies/rearrangements, deletions or duplications of multiple genes; study analysing embryonic/foetal but not female DNA; study with male patients only; study investigating disorders of sex development; study investigating severe genetic conditions where infertility is a minor manifestation of the condition; study not available in English.

B) Protocol used for gene-disease clinical validity assessment

Original protocol: “Classification of Genes: Standardized Clinical Validity Assessment of Gene–Disease Associations Aids Diagnostic Exome Analysis and Reclassifications” by Smith et al. (Smith *et al.*, 2017).

Follow the current protocol step by step and fill in the required information into the gene-disease curation table (Supplementary Table S3).

1. **Patients**. Screen the manuscript for the presence of female patients with certain female reproductive failure phenotype in an isolated form or in association with the particular syndrome. Count only patients having gene variant classified as a VUS or higher (see variant classification below). Count all unrelated female patients, count all female patients within a family as one (fill column ‘Number of unrelated patients’).
2. **Gene**. Open the gene in OMIM (https://omim.org/). Check whether it is assigned an identification number associated with the particular female reproductive failure phenotype under consideration in the article. Write down the number and the reported inheritance pattern (fill columns ‘OMIM gene:phenotype identifiler’ and ‘OMIM Inheritance pattern’).
3. Identify **inheritance pattern** reported in the manuscript (fill ‘Reported inheritance pattern’). For autosomal dominant diseases assign one point for the identification of a significant excess of *de novo* alterations in a large cohort study. For autosomal recessive inheritance assign one point if the LOD score is >3 (applicable to large families with multiple affected individuals) (fill ‘Other statistical evidence’, maximum 1 point).
4. **Variant classification**. Adhere to the ACMG gene variant classification guidelines (Richards *et al.*, 2015). Patients having likely benign/benign variants should not be counted. If manuscript describes only (likely) benign variants, mark the article as

‘unable to classify’. Assign one point for each likely (pathogenic) variant identified in the study (fill ‘Number of (likely) pathogenic variants’, maximum 4 points).

5. **Gene function**. Assign one point if the gene function/expression is consistent with the disease. If article is missing this information, lookup for the databases, e.g., Protein Atlas (https://www.proteinatlas.org/) or GTEx (https://www.gtexportal.org/home/) (fill primary organ/tissue of expression in column ‘Target organ(s)’). Assign one point if the gene physically interacts with another gene characterized for the same disease. If article is missing this information, lookup for the databases, e.g., String database (https://string-db.org/), (fill ‘Gene function’, maximum 2 points).
6. **Gene disruption**. Assign one point if relevant pathology is observed *in vitro* after similar genetic modification. Assign one point if mutational mechanism is determined. If article is missing this information lookup for the additional articles, (fill ‘Gene disruption’, maximum 2 points).
7. **Model organism**. Assign one point if the gene disruption *in vivo* is related to the pathology of human disease. Assign one point if model organism’s phenotype and genotype match human disease. If article is missing this information lookup for the additional articles or MGI database (http://www.informatics.jax.org/), (fill ‘Model organism’, maximum 2 points).

Repeat all the steps for all publications identified for the particular gene-disease association. Assign points for all patient number as follows: 1 point for 1-2 patients, 2 points for 3-4 patients, 3 points for 5-9 patients, 4 points for 10-24 patients (fill ‘Patient number score’).

Assign points for the replication studies, no point for the first publication, (fill ‘Number of replication studies’, maximum 3 points, and fill all curated articles PMIDs in ‘References (PMID)’).

After all publications are assessed, count the points for the gene-disease association and assign an evidence class:

<4 – No evidence;

4-8 – Limited;

9-12 – Moderate;

>12 – Strong;

If patients’ number is >24 – Definitive.
